# “Happiness because I could play again”: professional mother footballers’ experiences of pregnancy and postpartum

**DOI:** 10.3389/fspor.2026.1809328

**Published:** 2026-08-13

**Authors:** Margie H. Davenport, José M. Oliva-Lozano, Frankie Brown, Katie Kavic, Tara-Leigh McHugh

**Affiliations:** 1 Program for Pregnancy and Postpartum Health, Physical Activity and Diabetes Laboratory, Faculty of Kinesiology, Sport, and Recreation, Women and Children’s Health Research Institute, Alberta Diabetes Institute, University of Alberta, Edmonton, AB, Canada; 2 United States Soccer Federation, Fayetteville, GA, United States; 3 Sports Exercise and Health Science Research Group, Edinburgh Napier University, Edinburgh, Scotland; 4 Faculty of Kinesiology, Sport, and Recreation, University of Alberta, Edmonton, AB, Canada; 5 Faculty of Kinesiology, University of Calgary, Calgary, AB, Canada

**Keywords:** football, postpartum, pregnancy, professional, qualitative research

## Abstract

**Introduction:**

Women’s football is at a pivotal moment in both sporting and social discourse, with its rapidly expanding fan base making it on track to become one of the world’s top-five women’s sports by 2030. Yet as the sport’s commercial value grows, critical questions remain about how the game accommodates athletes’ life events. Pregnancy is of particular interest as the physiological changes during pregnancy necessitate medical and performance considerations that coincides with a historical belief that starting a family means retirement from sport. This study aims to fill a critical gap in research by documenting the experiences of professional mother footballers as they navigate pregnancy and return-to-football postpartum with the ultimate goal to provide a foundation for evidence-informed protocols to safeguard their health and professional aspirations.

**Methods:**

Qualitative description was employed as the methodological approach, as this design is well-suited for topics with limited existing research and prioritizes rich, plain-language representation of participant experiences. Findings generated through qualitative description are grounded in “everyday language” and are particularly relevant to policy makers and practitioners. 9 professional mother footballers participated in the study. Players were 38.6 ± 4.5 years of age and had 13.2 ± 5.4 years of football experience across three continents. One-on-one semi-structured interviews were utilized to explore the pregnancy and postpartum experiences of professional mother footballers. A three phase (preparation, organization, and reporting) process of content analysis was employed for data analysis.

**Results:**

Our findings revealed four interrelated themes that represent mother footballers experiences of pregnancy and return-to-football postpartum: (a) “It’s not one size fits all”: Progression away from gameplay, (b) “Gaining strength and easing back”: Return to gameplay, (c) “One step forward, two steps back”: Triumphs and challenges of mother footballers, and (d) Recent wins for women and opportunities for growth. Discussion: This study provides insights for sport organizations, federations, and any performance team supporting female football players who are considering starting a family mid-career. Findings suggest that maintaining both athletic and mother identity through the transition back to professional football are mediated by four distinct yet interrelated themes that challenge standard return-to-sport protocols.

## Introduction

Women's football is at a pivotal moment in both sporting and social discourse, with its rapidly expanding fan base making it on track to become one of the world's top-five women's sports by 2030 (1). Yet as the sport's commercial value grows, critical questions remain about how the game accommodates athletes’ life events. Pregnancy is of particular interest as the physiological changes during pregnancy necessitate medical and performance considerations that coincides with a historical belief that starting a family means retirement from sport. In response, FIFA has established minimum maternity protections within its Regulations on the Status and Transfer of Players (2021; updated 2024) and FIFPRO has actively championed maternity rights, and parental-leave safeguards to ensure that choosing to have a child does not force athletes to end their careers prematurely (2–4). While the FIFA maternity mandate represents a landmark institutional step toward safeguarding athletes’ reproductive rights, its initial practical implementation revealed significant regulatory gaps. Frequently, overarching directives lacked the specific mechanisms required to guarantee comprehensive, multidisciplinary care across varying club environments, leaving athletes vulnerable to inconsistent support. The development of FIFA's “Stay in Play” and “Stepping into Play” frameworks to support pregnant and postpartum athletes now actively seeks to address this gap but their implementation has not yet been tested (5, 6).

Extensive work has documented the experiences of elite athletes, Olympians and World Champions, during pregnancy and postpartum (7, 8), and such research has provided necessary insights into the unique challenges, and critical supports required, for elite athletes to navigate pregnancy and postpartum within inequitable sport systems. Pregnant elite athletes have described how they are hesitant to disclose their pregnancy to coaches, sport organisations, and sponsors, in fear of negative repercussions, including loss of position on their team and funding (9). Such fears are well founded, given the well-documented “motherhood penalty” that exists in sport (10). Becoming a mother restricts career advancement, earnings, and satisfaction, and such challenges are “exacerbated in sport” where the sport culture “marginalises mothers” (p. 120) (10).

Research grounded in the lived experiences of elite pregnant, postpartum, and mothering athletes has outlined the extensive support networks, including partners, extended families, and sponsors that are necessary for supporting their athletic careers (9, 11–13). Interviews with elite athletes have also highlighted how athletes experience an “identity crisis” in the transition from athlete to mother athlete. Darroch and Hillsburg (2017) described how some elite athletes have reported feelings of guilt when returning to training after childbirth, yet other athletes have described how training and competing enhanced their mothering, ultimately transforming feelings of guilt into those of empo werment (13). Recognising the complex ways in which athletes must navigate dual, and often conflicting, identities as mothers and athletes, various actionable steps have been identified by the athletes themselves (9). These athletes have strongly argued for better education, awareness and supports for both training and competition (9, 12). Sport organizations have begun to develop structural supports for these athletes but major gaps remain to ensure gender equity for athletes who are pregnant or parents (14).

To date research in this area has primarily focused on the experiences of amateur athletes and those of professional players remain relatively unexplored. Indeed, football is one of the few sport contexts whereby the experiences of professional mother footballers have received some attention in the published literature (15, 16). Findings from such research has shown that working conditions for mother footballers varies greatly between clubs, and professional football is a “field for women that is still emerging and adapting to their gender-specific needs” (15). Further, with FIFA's 2021 global mandate for all member associations to implement minimum maternity standards for players, the professional football landscape for women athletes has seen exceptional change and growth over the last five years (1). The complex experiences of mother footballers continue to be shaped by such broader socio-political mandates, providing strong justification for more research that is grounded in the distinct experiences of professional mother footballers. Such research is essential for understanding potential disparities in access to multidisciplinary medical teams and regulatory protections for mother footballers, as well as contracts and pressures for a rapid return to play.

This study aims to fill a critical gap in research by documenting the experiences of professional mother footballers as they navigate pregnancy and return-to-football postpartum with the ultimate goal to provide a foundation for evidence-informed protocols to safeguard their health and professional aspirations.

## Methods

This study received ethical approval from the University of Alberta Research Ethics Board (Pro00151952). Qualitative description was employed as the methodological approach, as this design is well-suited for topics with limited existing research and prioritizes rich, plain-language representation of participant experiences (17). Findings generated through qualitative description are grounded in “everyday language” and are particularly relevant to policy makers and practitioners. Qualitative description is a particularly useful methodology in sport research whereby researchers seek to develop an evidence-base that can inform decision-making by sport organizations (18, 19).

## Participants

Consistent with qualitative description methodology (17), purposeful and snowball sampling were used to recruit professional footballers who have been pregnant during their career (20). Eligible athletes were over 18 years of age, and were playing professional football at the time of conception in the previous five years. Recruitment occurred through the authors personal networks and word-of-mouth referral. This process of recruitment is essential for identifying potential participants from hard-to-reach populations or for research studies, such as this, that involve sensitive topics that could have detrimental outcomes if a participants’ anonymity is compromised (21).

Nine professional mother footballers, with professional experience playing in the United States or Europe, participated in the study. Players were 38.6 ± 4.5 years of age and had 13.2 ± 5.4 years of football experience (Table 1). To protect confidentiality, pseudonyms are used and any potentially identifying information has not been included.

**Table 1:** Participant characteristics.

Demographic	Mean ± Standard Deviation/% (*n*)
Age (years)	38.6 ± 4.5
Ethnicity	
White	78% (7)
Black	11% (1)
Mixed heritage	11% (1)
Football History	
Retired	44% (*n* = 4)
Football participation (years)	22 ± 4
Professional/International football participation (years)	11 ± 5
Level of play during pregnancy	
Professional	78% (7)
International	22% (2)
Difficulty getting pregnant	
No	89% (8)
No, but it took more than 6 months	11% (1)

## Data generation

The intent of a qualitative description study is to describe experiences from the “viewpoint of the person(s) in the midst of the experience.” (17) Accordingly, one-on-one semi-structured interviews were utilized to explore the pregnancy and postpartum experiences of professional mother footballers. Participants first provided written informed consent and completed a brief questionnaire describing their demographic characteristics, including their general sporting background, professional football experience, and details about their pregnanc(ies).

A woman research assistant (KK), trained in qualitative research methodologies, facilitated all of the one-on-one interviews with the participants. The semi-structured interview guide was informed by football and pregnancy research, developed by the research team. The research team has professional expertise in qualitative sport research and applied sport science, including team members who work with professional football and international sport organizations. Relevant to the study, the research team also has lived experiences as high performance athletes, coaches, and mothers. The semi-structured interview guide included 10 main questions, each accompanied by an average of three follow-up and probe questions, that sought to address the study's aim to document the experiences of professional mother footballers as they navigate pregnancy and return-to-football postpartum. Given the exploratory nature of the research, the questions were intentionally broad to support participants in sharing their experiences related to the broad range of biopsychosocial considerations that shape athletes sport participation during pregnancy and postpartum. Three general types of questions were included in the interview guide, including *introduction* questions (e.g., “How did you get involved in football?”), *focused* questions (e.g., “How long did you continue to train during pregnancy?”; “What do you know now that would have helped with your training during pregnancy and/or return to football?”), and *closing* questions (e.g., “What have we not discussed today, but you think is important to share with respect to the experiences of pregnant or postpartum footballers?”). The semi-structured nature of the interviews enabled clarifying or probing questions when participants described key experiences.

All interviews were conducted in a manner that was responsive to each participant's comfort and communication preferences. Interviews were conducted via secure videoconferencing platforms and lasted between 48 and 72 min (average of 60 min; total of 542 min across all 9 participants). Interviews were audio-recorded, transcribed verbatim using Otter.ai (2024), and checked for accuracy by the research team. A total of 279 pages of single-spaced transcripts were generated from all interviews. While participants did not review full transcripts, they were provided with the opportunity to follow-up with KK after the interviews to provide clarification on topics discussed (including deletion of specific comments) and/or to add additional context to discussion; none of the participants provided additional follow-up. Immediately following each interview, the interviewer created narrative summaries to support early familiarization and collaborative interpretation across the research team.

## Data analysis

The goal of qualitative description is to stay “data near” and to provide a clear, straightforward account of participants’ experiences using their own language (17). As such, a three phase (preparation, organization, and reporting) process of content analysis was employed (22).

In the preparation phase, transcripts were cleaned and all identifiers were removed; participant names were replaced with pseudonyms to protect anonymity. The transcripts and narrative summaries were read repeatedly for immersion and to gain a “whole” sense of the data (22). Consistent with a qualitative descriptive approach, the manifest content of the data (ie., words) were used as the primary unit of analysis. The organization phase involved structured open coding, whereby the interviewer independently recorded notes and headings in the transcript margins in an effort to describe all aspects of the content. Headings were then used to generate categories; categories and the supporting codes were used as the basis for analytical discussion among KK and two additional research team members (MD, TM). Through this in-depth discussion, categories were collapsed and higher order themes were generated to represent the findings.

In the reporting phase, themes were described using participants’ everyday language, and illustrative direct quotes were selected to highlight the shared and unique experiences of mother footballers. This process of reporting findings is essential for ensuring research respectively represents the voices of lived realities of participants.

## Results

Four interrelated themes represent mother footballers experiences of pregnancy and return-to-football postpartum: (a) “It's not one size fits all”: Progression away from gameplay, (b) “Gaining strength and easing back”: Return to gameplay, (c) “One step forward, two steps back”: Triumphs and challenges of mother footballers, and (d) Recent wins for women and opportunities for growth. Each theme is detailed in the following sub-sections and supported by direct quotes to illustrate the depth and complexity of participants’ experiences.

### “It’s not one size fits all”: progression away from gameplay

Mother footballers shared in-depth insights into the broad range of physical, mental, emotional, and social changes they experienced during their pregnancies, and how such changes were intricately intertwined with their progression away from football gameplay. While there were some commonalties in these experiences, participants were clear in their contention that pregnancy is an individual and personal experience, and no two pregnancies are the same. As stated by Emma, “No pregnancy is the same. No individual is the same. We all go through different things and different experiences. Don't compare what you're going through because it is your personal experience. It's not someone else's.” Similarly, when referring to the FIFA maternity policy, Laura stated, “I think 14 weeks minimum maternity is great. It's a good start. But I think every pregnancy is so different, and it's hard to put a timescale on it, because some people might be back within that 14 weeks, some people might need a lot longer.” -Susan

Participants detailed diverse timelines with respect to when, and how, they progressed away from gameplay during pregnancy. Unlike the majority of mother footballers, two participants explained how they stopped all gameplay almost immediately after confirming their pregnancy. Kathryn explained, “At 10 weeks I just had such bad sickness, I went back to my club and just said ‘Well, I can't train, I just can't keep anything down’…literally through my whole pregnancy it was another thing (sickness) after another, which prevented me from training a lot.” Unlike Kathryn, Nasim was physically well enough to play during her pregnancy, but mentally she was unsure. She said, during pregnancy, I was at an interesting point of, ‘Do I want to continue training? Do I want to continue playing soccer after? So I was just kind of trying to figure out all those things.so I just kind of stepped away from the game for a bit… But I ended up working out, running and lifting– I should be specific, not really playing soccer at all, but I ran and lifted up until I think week 38 of my pregnancy, and just kind of stepped away from any soccer things.

Participants generally explained how they engaged in gameplay until they were 12–14 weeks pregnant, and then they moved to non-contact gameplay with their team. However, there was great variability in terms of mother footballers training specificities and timelines during pregnancy. As explained by Anne, “The doctor that looks after me made sure everything's okay, and the physio actually wrote me a program and I trained up until 6 months with the team.” Helen, who had previously experienced a miscarriage, explained how during her subsequent pregnancy, she made the decision not to engage in any contact. She said,

I was still allowed to train, albeit I made the decision to go straight away into non-contact training. And so it was just very much like running, passing the ball. There was no tackling, but that was all my own personal decision, just from my past experiences.

The diverse timelines of when and how participants moved away from gameplay during pregnancy provides further insight into mother footballers’ argument that the physical, mental, and emotional experiences of pregnancy are unique among all individuals.

Despite all participants moving away from gameplay during their pregnancy, each mother footballer described how they continued to train throughout their pregnancy by engaging in ball and running drills on the sidelines of the pitch, or participating in other activities such as biking, running, and swimming. Anne explained that when she got to the “point where I felt uncomfortable [6 months pregnant], or I felt my center of gravity was changing…that's when I moved to going to the gym. It might be biking, swimming. I absolutely loved swimming” -Anne. While training was acknowledged as an important aspect of maintaining their fitness during pregnancy, some participants also expressed how training was important in managing their weight gain. Ivi shared, “As athletes, you are never more than a few kilograms up or down with your weight, so putting on weight was difficult…it was mentally harder to take.” Similarly, in referring to weight-related discussions she had with her doctor during her pregnancy, Nasim said:

Oh, my gosh! I feel like this is too much! And my doctor was like, ‘no, you're doing it perfectly. You're by the textbook’…I mean, just as an elite athlete you don't want to be gaining 20 extra pounds…it was like a little nerve wracking. But my OB reassured me.

This notion that elite athletes fear weight gain, even during pregnancy, was further explained by Anne who said,

I didn't want to gain a lot of weight. One thing in my life is I’ve always been terrified of gaining weight, and even then [while pregnant] it would be tiring, I still had that fear and needed to train. I don't want to gain weight. I think it's just something that has been drilled into me, because I've played sport from such a young age.

Whether driven by their motivations to maintain fitness levels, or to specifically manage their weight gain, most participants described how they engaged in some level of training throughout their pregnancy.

### “Gaining strength and easing back”: return to gameplay

Strength and conditioning were described by mother footballers as a central component in their gradual return to gameplay postpartum. Pelvic floor and core strength were acknowledged as being particularly important for their return. Ivi returned to football one month after her child was born and she shared: “In the beginning I was doing all pelvic floor exercises and just simple, straight line running to ease my way back into fitness.” Similarly, in referring to her return to gameplay, Nasim explained, “the strength coach kind of helped to ease me back in from his side of things, and he was very conscious of my pelvic floor.” Susan described how her body did not “feel right” when she began running about six or seven weeks after childbirth. She said, “Everything felt loose, and the physio said, ‘Have you got diastasis recti?’ It sounded like a foreign language to me…I had to do a lot of work on my deep muscles to get them back together.”

Despite the challenges they faced with their physiological readiness to play postpartum, mother footballers shared detailed accounts of how their gradual return to gameplay generally had a positive impact their overall mental well-being. Ivi explained,

Mentally, it [return to gameplay] was good. I felt like I was just back, and it was nice. Physically, it was hard, because I felt very unfit, and I've never been unfit for football. But obviously it came in time, and I was fine after a few months.

As high-performance athletes, being part of their team played an important role in supporting their mental well-being. Participants, like Ivi, took a brief break away from their team during pregnancy or postpartum. Participants like Nasim, however, engaged with their team throughout their pregnancy, noting “It was great to still be in the team setting, even though I wasn't on the field.” As shared by Patricia, “having the option of being connected [to the team] in the way that you want to be connected” is so important for athletes. Reflecting on her return to gameplay, Sinead shared:

I mean, the first game was just like happiness because I could play again. I've been waiting for so long I thought, and I remember that my body felt quite okay with me. I haven't played for like 16 months or something with the pregnancy and everything, so it was a long time ago. But I remember I was just so happy.

Regardless of whether the participants took a leave from the team, or continued to engage, participants described how the guidance they received from support staff (e.g., physicians, physiotherapists, trainers) played a critical role in supporting their mental readiness for return to gameplay. As shared by Emma, “what I have found extremely powerful and helpful is when the medical team and coaching staff connect…because when there is not communication there is stigma around what we can do” and that is stressful for the athlete. Anne further explained, “My doctor, and the Physio helped me mentally because I knew what I had to do…because they guided me.” Participants also described the critical role of pelvic floor physiotherapists or pelvic health specialists in supporting their timely return to gameplay. As articulated by Nasim: “So my pelvic floor PT recommended waiting 12 weeks before I start jogging again, which sounded crazy to me, but I let her lead the way.” Sinead explained how she returned to her team two weeks after childbirth but she mostly “did training by myself but with the physio and strength coach.” With the support of her support team, Sinead explained how she began light jogging six weeks after giving birth, which is notably different guidance than that received by Nasim.

While many mother footballers described how the guidance they received was important for supporting their physiological and psychological readiness for returning to gameplay, not all mother footballers received such guidance or support. Sinead shared, how she received little guidance and was simply told to “just listen to your body”. She further explained,

I did a lot of stuff by myself. I ran when they played games, and I just tried to build up my body again. It felt like I waited for my body to get ready to do everything on the pitch again.

For Helen, she explained how she was one of the first in her organization to have a child and therefore there was very little support. She said, “I had to decide what was best for me. It was very much an unknown area, like nobody really knew the best way forward. So it was very much just up to me.” For mother footballers like Sinead and Helen, the lack of guidance served as an impetus to stress with their return to gameplay.

### “One step forward, two steps back”: triumphs and challenges of mother footballers

The detailed stories shared by participants provide unique insight into the triumphs and challenges experienced by mother footballers when they return to their sport after childbirth. As noted in the above theme, mother footballers described how their return to their team setting and gameplay was important for their mental well-being. However, as their training progressed upon their return to their sport and they described their physical gains, these stories were also intertwined with experiences of various physiological challenges. As shared by Sinead,

Every time when I started to do harder runs, more changing directions, I felt this strange pain in my groin again, and I couldn't really run as much as I wanted to…I felt like it was hard to make the decision– is it okay with the pain I have? Can I push it through? Or should I take a few steps back again? I struggled a lot with that…it felt like two steps back all of the time.

In addition to pain, participants like Kathryn noted how she experienced challenges with her balance. She explained:

I'd been doing straight line running, but I never really took into consideration you're running at all different angles in football, so that put me a step back. And it's sort of knocked my confidence a little bit. I was like, ‘Oh, my God! Am I ever going recover?’

Participants also described their experienced mental struggles when their gameplay was not at the same level as it was prior to pregnancy. As stated by Sinead, “I think mentally, I was struggling quite a lot with this part, like coming back to full training again, like at some point I thought like, can I even play again?” Similarly, Kathryn explained,

I sort of lost interest in the game. I thought, ‘oh well, I'm not back to my best’. I didn't really get any support in terms of a training program you need to follow, and what I need to do if I want to get back to my best. And I just started to lose interest, really.

Susan also shared, Psychologically I struggled with not being able to do what my mind was telling me to do, like you try and make a pass or move your feet, and they're just not used to moving that quick. So my mind was ahead of my body, if that makes sense.

As articulated by Sinead, Kathryn, and Susan, high-performance athletes may lose interest and/or experience mental struggles when they do not perceive their performance has reached the level it was prior to having a baby.

The psychological challenges that mother footballers described were not just related to their performance, but in part due to the need to navigate their new identity as mother footballers. In describing her return to gameplay when her child was four months old, Kathryn explained how her priorities shifted, particularly as they related to international play:

At 4 months old I wouldn't feel ready to just leave my baby and go away on international duty, I said. I don't know whether it's like a motherly instinct, I wouldn't want to be away from my child that long…So it's [gameplay] is not something that was burning inside me…So I said I can't imagine being away from my child that long, even a night, let alone a week at a time.

Anne also explained how mentally difficult it can be when trying to balance responsibilities as a mother and a footballer. Referring to when she had her first child, Anne shared,

It was quite a lonely time within the women's game with having a child, no one really did it. I think for that reason, there was no support. I live with my children day in and day out, and they wake me up in the middle of the night, the morning, and that's how my life is, that's how I live.

Somewhat contrary to what the other mother footballers shared, Helen explained how becoming a mother made her a better player. She explained,

I felt like a better player, and purely down to the fact that I could switch off from the sport when needed, whereas before pregnancy and before having a child my sole focus was the sport. And this very much wraps you up in that, and it consumes you. Whereas when you have that child to return home to, you can switch your mind off it. So when you do go into training, or you do go into a game, you appreciate the time, because that's your time to solely focus on your sport for the few hours that you're there, and then you can return home and go back to normal life.

### “Recent wins for women”: and opportunities for growth

This final theme represents shared stories of notable progress that mother athletes, and footballers in particular, have achieved within high-performance sport contexts. By describing their own detailed accounts of mother footballer experiences, various areas of growth were also illuminated. Most notably, participants acknowledged the FIFA maternity policy and how critical this policy has been for supporting athletes. Susan shared, “It's good that there is a policy, and it gives players that sort of sense of security… it gives players a bit more peace of mind.” Also referring to the maternity policy, Anne said, “I think the support is becoming better. I think we’ve seen improvements across the board… I think we're heading in the right direction in terms of supporting females through pregnancy and back onto the grass.” As argued by Emma, knowing that athletes have this level of policy support is essentially for being able to “plan strategically”. However, despite the progress, Emma stated, “I do think there is still discrimination that exists against athletes who want to get pregnant.” Furthermore, Patricia described the critical need to ensure that “there's a lot of flexibility worked into policies, because everybody's experience is very different”.

In addition to maternity policies, participants also explained how there is generally more medical support for professional mother footballers than there was in the recent past. Sharing the story about when she announced her pregnancy to her team, Sinead said: “I was just happy that the club was like, ‘Okay it's fine if you're pregnant, we're gonna support you and let us know if you need anything’….I had good physios and doctors and stuff like that”. Similarly, Nasim shared, “I had a lot of support between my pelvic floor physiotherapist and from [club] trainers sending workouts and things like that.” Despite perceived good intentions to provide support, some participants explained how some support staff acknowledged shortcomings in their ability to do so. Kathryn said,

I spoke to the physio, and they were predominantly men and I remember him just saying to me, ‘I'm going to be totally honest with you, I've never worked with someone that's pregnant, and I'm not really sure what advice I should and shouldn't be giving you.

Despite the majority of athletes describing some level of support, two participants explained how they were the first women to have babies in their respective football club, and they received little guidance on how to train during pregnancy and postpartum. Instead, to guide their training, they were simply told to “listen to their bodies”. As described by Ivi,

I think I could have done more while I was pregnant. So I think I got to a stage where I was just like, ‘Oh, I'm pregnant, now I can't do anything’. But I probably could have. There was noone telling me what to do or what not to do, so I just had to go with what I thought was right. Looking back I could have done more.

Building upon this notion of wanting to “do more” in terms of training during pregnancy and return to sport postpartum, most participants described the importance of having “female health specialists” as part of any training team. Helen specifically noted the importance of having a “pelvic floor specialist”, particularly to support return to play. Similarly, Kathryn argued for having a support team member that “actually knows female anatomy and what can and can't happen, and what does happen during and after pregnancy.”

Participants also described the need for mandatory education for athletes, coaches, and support staff, particularly in regards to how mothering impacts the psychological well-being of mother footballers. As argued by Emma, *“*training and education should be mandate because to me, this is safe sport.” Patricia went as far as arguing the need for “training on a yearly basis”, so that mother footballers can be supported. Indeed, all participants argued for more access to information and support on evidence-based best practices for training during pregnancy and postpartum. While most participants acknowledged that training supports have increased over the last few years for mother footballers, they argued that this is not the case for psychological or mental supports. In referring to becoming pregnant after a miscarriage, Nasim said: “I guess mentally, I just didn't want to push it too hard. After going through that, and then emotionally, I didn't want to have to go through that again.” While it is recognized that Nasim's miscarriage likely facilitated additional stress about training during pregnancy, other participants who did not experience miscarriage also shared their worries about how training during pregnancy might be detrimental to their babies’ health. As well, the mental struggles of returning to sport that were described by participants mother footballers served as an additional call for psychological supports during the postpartum period. Susan shared, “Psychological support I'd say that's arguably more important than physical support. I found I was always having a sense of guilt around going back to football and leaving my kids with grandparents…and whether I should have carried on playing.”

## Discussion

Historically, professional football regarded pregnancy and elite performance as mutually exclusive, often leaving players without contractual protection, training supports or financial security. However, this perception has undergone a significant shift in recent years, catalyzed by the growing number of football-mothers defying gendered expectations and the 2021 implementation of FIFA's first global maternity regulations (2). While the implementation of these policies represent a critical move toward ensuring the rights and protection of mother footballers, the lived experiences of players continue to reveal a complex tension between these new policies and the physical and cultural realities of returning to the pitch.

The findings of the current study suggest that maintaining both athletic and mother identity through the transition back to professional football are mediated by four distinct yet interrelated themes that challenge standard return-to-sport protocols. Mother footballers shared their lived experiences of attempting to balance physical and psychological readiness to return with club expectations on time to return. Players highlighted the essential need for individualized progression during return-to-play, rather than the current “one size fits all” expectations given that the postpartum return-to-sport journey is highly individual, non-linear, and influenced by numerous physical, psychological, and social factors. They highlighted that current time-based expectations created unrealistic expectations as no two pregnancies, births, or recovery are identical. Rather, most experienced a non-linear progression as they navigated return to professional football.

The experiences shared by professional mother footballers in this study contributes to a growing body of research that highlights the complexities of navigating pregnancy and parenting within elite sport (12, 15). Consistent with previous research focused on elite athletes and Olympians of various sports, mother footballers described how they navigated fears of losing their position on their team, and they documented an identity crisis during the transition to a mother-athlete. However, the team-based nature of football offers a unique social context where participants emphasized the importance of remaining connected to the team to maintain their professional athlete identity, a support mechanism that may be less accessible to athletes in individual sports. While participants acknowledged that the implementation of FIFA's maternity regulations was significant in protecting their rights to start a family, the persistent barriers outlined by players underscore an urgent need for evidence-based, football-specific frameworks. To operationalize these findings and support the long-term careers of mother footballers, sport organizations, federations, and performance teams should consider the following practical recommendations (see Table 2).

**Table 2:** Practical applications to support pregnant and postpartum players.

Themes	Example supporting quotes	Policy recommendations and actionable steps to support players
“It's not one size fits all”: Progression away from gameplay	*I think the social side of it. So being a part of that team like still going along, even when your 34, 40 weeks pregnant that you still got that team around you that you've always had like rather than segregating yourself off, because you're pregnant that you should still be involved in the team, and I think that helped me massively.* *Looking back I could have done more, and I wish I did do more, so I wouldn't have been as unfit as I was, obviously it's different because pregnancy is not an injury, but I feel like I could have done more.*	Recognize the importance of modified gameplay to support continued technical/tactical training, team inclusion and athletic identity. Support collaborative, player-centered communication that enables and empowers players in their decision-making. Provide education and specialized supports (e.g., pelvic floor specialists, mental health, and sport scientists) that can help support pregnancy-related changes in physical and mental health.
“Gaining strength and easing back”: Return to gameplay	*Maybe like the weight gain and stuff like that in pregnancy, because obviously, as athletes, you are never more than a few kilograms up or down with your weight, so putting on 3 stones or 2 and a half stone was difficult. Obviously it came off afterwards, but it's just that knowing of your weight, which was mentally harder, I think, to take.* *There was nothing telling me what to do or what not to do, so I just had to go with what I thought was right…*	Develop individualized rehabilitation and training programs to support return-to-play. Include specialized supports for pelvic floor and psychological readiness. Normalize that progression will not be linear and regression is part of the process not failure.
*“One step forward, two steps back”: Triumphs and challenges of mother footballers*	* As I said to you, it's all individual, but if they’ve got a guideline or a structure to work from, you can push past that we can change and adapt things if the person's more ahead than they should be– like you can do all that stuff, but it is very personal. It's about how she feels on any given day, yeah.* *But being at [club], they had [name of player] at [club] who was pregnant while playing and they did a great job cause they hired people from outside in. So there's a pelvic health specialist that came in to help educate them on what needs to be done and what she needs.* *I think more information readily available for right at the beginning, because I didn't know anything when I think I found out when I was about 6 week and trying to find out what to do at that point, there was nothing I could turn to, anyone really to ask for what to do with football…even though I googled everything.*	Develop clear, individualized protocols that recognize the variability in athletes’ pregnancy and postpartum timelines. Emphasize functional milestone rather than fixed timelines in readiness-based frameworks, which may integrate physical, psychological and other contextual indicators (e.g., pelvic floor function, load tolerance, movement quality, psychological readiness, and other contextual factors such as sleep disruption, childcare demands, lactation, etc). Foster an inclusive club/federation culture where motherhood is valued and viewed as compatible with high-performance football.
“Recent wins for women”: And opportunities for growth	*A lot of clubs now have doctors and sports scientists, physiotherapists, a lot of them employ somebody that is a specialist in [women's health and performance], and I think now with the maternity laws in place, particularly in the [league] and the championship clubs will support their players through their pregnancy, and put them on a tailored program throughout and then again, once they return to play.* *Now, that's what I say to you, if a female feels supported within pregnancy and coming back and getting back onto the pitch, they'd be more open and honest with how they feel and how it's going. And I think there'd be less pressure on them to return to play, because I’ve heard stories of people feeling under pressure in terms of whether it be sponsors that are trying to get them back because the brands, and there's all those sorts of pressures. But you know, we put pressure on ourselves anyway, as a female and as an athlete, to get to a certain level. But yeah, I think support is a big one.*	Strengthen and enforce regulatory protections beyond FIFA maternity policy. Create and share national guidelines for perinatal player support. Offer education programs for coaches and other training opportunities for medical staff and administrators on player pregnancy and postpartum care. Implement mandatory bias-awareness training for technical commissions and coaching staff.

### Individualized, readiness-based return-to-play

A key application was the players’ advocacy for clubs to shift from utilizing weeks postpartum as a primary marker for readiness to individualized frameworks that focus on their unique, non-linear progression that would provide a more realistic return-to-play expectation. While some players suggested that existing time-based frameworks were too conservative and they felt ready to return sooner, others found the progression too rapid creating un-necessary stress. Examples of good practice exist to mitigate this undue stress, both at individual club level and league wide. In Italy, the professional club AC Milan introduced a new policy in 2024 that guaranteed an automatic 1 year contract extension for female players who are pregnant in the final year of their contract (23). While the 2024 collective bargaining agreement (CBA) that applies, league wide, to the National Women's Soccer League (NWSL) in the USA allows flexibility for return-to-play with no fixed ‘weeks postpartum’ stated, players will receive full pay and insurance cover for the whole duration of the return-to-play (30). Recently, in 2025, the Norwegian womens football league, the Toppserien introduced a league wide policy, granting an automatic 1-year contract extension (with the same terms) to any players who reach the 12th week of pregnancy, before the end of their last contract. These new policies/contracts were implemented to ensure that players have the opportunity to return to gameplay on an individualized timeline.

Similar to recently published expert reviews, players further argued for a more holistic assessment of health to consider pelvic floor function, musculoskeletal load tolerance, and movement quality, along with the unique contextual demands of early motherhood, such as sleep disruption, the physiological requirements of lactation, and the logistical challenges of childcare (24, 25). To operationalize these findings, football clubs should prioritize the integration of specialized expertise into their medical and performance departments. This includes formalizing consultations with pelvic floor physiotherapists and sports psychologists who possess specific expertise in the intersection of motherhood and elite performance. The release of FIFA's “Stay in Play” and “Stepping into Play” frameworks to support pregnant and postpartum incorporates these considerations (5, 6). Validation of these tools is essential. Ultimately, clubs must strive to foster an inclusive culture where motherhood is viewed as a compatible component of a high-performance career rather than an obstacle to it.

### Adapting a wholistic approach to supporting players

On a systemic level, governing bodies and federations play a vital role in establishing the minimum standards of care (14). While existing FIFA maternity policies provide a necessary legal foundation, federations and clubs should aim to strengthen these protections through the creation of detailed practical guidance. This involves developing educational programs for coaches, medical staff, and administrators to ensure that the support system surrounding the player is informed and supportive. By recruiting pelvic health specialists and providing access to mental health professionals familiar with the elite sporting context, federations can ensure that the health of the player is protected regardless of the specific resources available at their individual club.

### Systematic change in supporting training during pregnancy

Coaches and performance staff are encouraged to redefine their approach to training during the perinatal period. Many players experienced being told to stop or drastically reduce their training. Removing players strongly affected athletic identity, and this conservative approach is not supported by current evidence-based guidelines for sport participation in pregnancy (24–28). Furthermore, there is a relatively strong body of research that is grounded in the lived experiences of elite women athletes, which has documented the psychological distress that can be experienced by athletes when they are forced to cease training as this is essentially forcing athletes to “abandon” a key aspect of their identity (11). Rather than focusing on outdated beliefs that pregnancy as a period of rest and relaxation where maintained participation is discouraged, extensive research demonstrates that pregnancy should be framed as a period when continued participation in sport is strongly encouraged (24). Planned, supervised, and gameplay that is modified when appropriate can include continuation of football drills such as ball work and small sided play can preserve technical and tactical engagement in football while maintaining the player's professional identity (24, 25).

Continuation of football-specific aerobic and resistance training programs are essential to maintain fitness and reduce injury postpartum. Research in other sports have demonstrated that continuation of training, even high-intensity and heavy weightlifting during pregnancy is central to maintained health and performance (29–34). Central to this approach is collaborative, player-centered communication with her coaches, performance staff and healthcare providers that empowers the player in her own decision-making, while proactively addressing potential pregnancy-specific concerns through evidence-based education. In 2024, FIFPRO produced a document outlining key considerations and essential supports to optimize support for postpartum players (4). This document highlighted the need for specialized supports (e.g., pelvic floor physiotherapist, lactation consultant, mental health), along with dedicated lactation rooms and other recommendations to ensure that players felt supported and a valued member of the team. Although a critical first step, players in the current study argue for the development of football-specific frameworks that provide practical recommendations on *how* to return to play. Players emphasized their desire for protocols that reflected the unique needs of the postpartum period (poor sleep, recovery, physical and mental vulnerability), rather than time-based protocols that did not account for the non-linear progression to gameplay postpartum that most participants experienced.

The current study complements previous work exploring the experiences of athlete mothers from elite-level amateur sports (7, 8). While extensive research suggest that the dual identity of mother and athlete can be combined, doing so often comes with cultural and financial challenges. Unlike many amateur sports, clear maternity regulations have been developed and refined for professional footballers outlining the duration and amount of funding, how pregnancy and parental leave would impact their eligibility to return to the team, as well as policies requiring individualized planning for training to support athlete wellbeing (14). However, similar to high-performance amateur athletes, the professional players in our study identified the lack of evidence-based information guiding return to football, as well as the inclusion of specialized supports including lactation, pelvic floor health and mental health services. Based on this work, key steps to improve the experiences of professional players through the motherhood transition have been identified.

### Mitigating unconscious bias

Beyond physical training modifications, it is crucial to address the role of the coach's subjective perception and potential ‘unconscious bias.’ Even when an athlete has reached physiological readiness, coaches may subconsciously underutilize mother footballers due to outdated gender norms or a ‘benevolent’ but harmful assumption that they are less committed or more fragile. This underutilization can limit a player's exposure and career progression. Therefore, education for technical staff must move beyond physiological safety to include bias-awareness training, ensuring that selection decisions are based on objective performance metrics rather than assumptions about a player's parental status.

The primary strength of this study is the identification of essential supports to facilitate a sustained career in professional football through pregnancy, postpartum and beyond. The use of in-depth interviews provided nuanced insights from professional players across three continents who experienced pregnancy within the last five years. Importantly, the findings should be interpreted through the lens of a potential ’survivorship bias.’ As our sample is comprised of nine professional mother footballers who successfully navigated the return to elite play, their narratives represent a specific trajectory of resilience. The profound identity conflicts, psychological distress, and logistical hurdles reported by these participants likely contribute to forced retirement for many other players. For athletes navigating this transition with less robust institutional support or within precarious contractual environments, the dual career of athlete-mother may be structurally unsustainable.

While this study provided a foundational look at the experiences of pregnancy in professional football players, it is not without limitations. Information regarding institutional support, contractual and financial conditions, specific medical guidance received, childbirth characteristics, and details of postpartum recovery was not collected. Given the relevance of these factors for interpreting athletes’ experiences during pregnancy and their return to sport, future studies should explicitly seek to explore these variables to better understand how they shape athletes’ decisions, experiences, and return-to-play processes. Furthermore, while numerous countries were represented, the majority were from Europe potentially limiting the generalizability of findings to regions with different cultural, social or club norms. The publication of new football-specific frameworks by FIFA for pregnant and postpartum players may help support players in less-resourced settings to return to play but have yet to be tested in a real-world setting. Furthermore, while these findings reflect relatively well-resourced environments, they primarily represent a European or North American perspective.

The absence of robust professional structures in regions like South America or Africa may further complicate the practical implementation of FIFA's maternity mandate. Future research must prioritize these diverse contexts to ensure maternity protections are globally accessible and enforceable for all athletes. Additionally while all participants identified as women, we recognize that this is a heterogeneous category, future work must explore how intersecting identities shape the challenges athletes face. For instance, the experiences of athletes from the Global South may differ significantly from the current sample (35). Further studies examining the impact of institutional support (access to financial and staff resources) may also contextualize the experiences of pregnancy and return to play postpartum. Addressing these gaps is essential for developing inclusive policies that reflect the true diversity of the sport.

## Conclusion

The findings of this study reveal that professional footballers navigate a non-linear return to play. These athletes often experience a profound identity conflict and psychological distress when rigid, time-based protocols fail to account for the non-linear complexities of postpartum recovery. Furthermore, the persistence of a motherhood penalty in professional contracts highlights a significant gap between international mandates and the actual multidisciplinary support available at the club level. To ensure motherhood is treated as a valued phase of a sustained career, organizations must shift toward individualized, readiness-based frameworks that accommodate the heterogeneous needs of athletes while providing the financial and contractual security necessary to pursue dual career and family goals.

## Data Availability

The original contributions presented in the study are included in the article/Supplementary Material, further inquiries can be directed to the corresponding author.
